# Progress in Psychiatry

**Published:** 1901-05-11

**Authors:** 


### ROGRESS IN PSYCHIATRY.

(*Continued from page* 85.)

**Mental Disorders following Syphilis.**—Syphilis is capable of producing, in those affected with it, mental and nervous disorders of the most varied kinds. For convenience a distinction may be drawn between congenital and acquired syphilis. Moreover, it may be pointed out that the offspring of syphilitic parents tend to exhibit various grave types of cerebral disorder and defect—idiocy, imbecility, epilepsy, congenital weakmindedness, and juvenile general paralysis—ascribable to the parental syphilis and dependent upon some form of malnutrition affecting the germ or sperm cells, or the embryo, during intrauterine life. It is interesting to note that analogous conditions may prevail in the offspring of alcoholic and phthisical parents, thus clearly showing the importance of healthy parentage and the deleterious results which may follow when grave malnutrition—whether from alcoholic, syphilitic, or tubercular sources—affects the embryo. The present chapter will deal only with the insanities and cerebral disorders dependent upon acquired syphilis. Syphilitic encephalopathy may follow recent infection,[82] or may make its appearance many years after the occurrence of a chancre.[83] Early syphilitic encephalopathy is due to the active ravages of the virus and is marked by vascular changes (endarteritis) and degenerative changes in the cerebral neurons. To these may be superadded neoplastic growths, whether cranial, meningeal, or intracerebral in situation. The encephalopathy which follows many years after syphilitic infection has been called by Fournier parasyphilitic. It is not marked by granulomata, gummata, or neoplastic products. Syphilitic encephalopathy is microbic in origin, whereas parasyphilitic encephalopathy is more complex in its ætiology, and is probably the net result of various obscure toxic conditions which have not as yet been elucidated. The following tabulation will serve to systematise facts regarding acquired syphilis, and to show broad distinctions which have a clinical and pathological importance:

| | | |
|---|---|---|
| *Active syphilitic diseases.* | Cerebro-spinal syphilis | Meningitis. Arteritis. Encephalitis. Myelitis. |
| | Peripheral neuritis | |
| *Parasyphilitic diseases.* | Paralytic dementia. Tabes dorsalis. Epilepsy. Neurasthenia. Hysteria. | |

Acquired syphilis actively affects the nervous system by specific lesions in a considerable proportion of all infected cases. Leaving the parasyphilitic group of diseases for the present out of the question, it may be stated that of all syphilitic cases which present tertiary symptoms 21 per cent. show involvement of the cerebro-spinal axis (Fournier). The cerebro-spinal lesions are widespread and somewhat irregular in distribution, and dependent as a rule on gummatous, chronic inflammatory, or neoplastic processes.

Cerebral syphilis, affecting more or less the mental functions, is only considered in this section. It occurs most frequently or most prominently in the form of a basal meningitis and arteritis starting from the circle of Willis and spreading laterally to the convexity of the frontal and parietal lobes of the brain (anterior and middle cerebral vascular areas). So commonly is this basilar region affected that, according to Charcot, it may never be considered exempt, even when the symptoms point clinically to lesions only of the convexity of the hemispheres. The meninges about the circle of Willis and the optic chiasma are infiltrated with small soft connective-tissue cells (plasma cells and fibroblasts) and the pia-arachnoid meshes are filled with viscoid lymphatic exudate and leucocytes. The adjacent nerve-sheaths (optic nerves, oculo-motor and facial nerves) may suffer in this process, while the basal vessels themselves also exhibit endarteritis obliterans in varying degrees. Neoplastic masses (gummata) may also develop along the blood vessels and nerves, and either superficially or deeply within the cerebral substance. The diffuse patches of meningo-arteritis as well as the gummata are liable, at a later stage, to undergo sclerous or fibroid changes. Large gummata may undergo caseation. The brain substance (cortex, white matter, basal ganglia, nerves) supplied by vessels involved in the morbid process or affected by the gummata will undergo degeneration and even softening, while at the margins of the affected areas a zone of encephalitis may often be recognised. Endarteritis may be a primary condition, or may be secondary to gummatous meningitis. The basal vessels frequently suffer from endarteritis, and the basal ganglia suffer very often and in like degree (Heubner). The middle and anterior cerebral arteries frequently show more or less obliteration of their branches and twigs. The weakened vessels are liable to undergo aneurysmal dilatation or rupture, and this is most commonly at the basal region of the hemispheres (lenticulostriate arteries). The involvement of cranial nerves may lead to ptosis (fibres of the oculo-motor nerve), pupillary stasis and inequality of the pupils, optic neuritis with irregularities of the visual field (optic chiasma and tracts), trigeminal neuralgias and facial palsies of temporary and fungacious character, and temporary amblyopias. The mental distress caused by these troubles may be and often is aggravated by severe headaches, cephalic paræsthesiæ, and insomnia. The headache is intense and severe, grinding or boring, deep-seated, and associated with local tenderness of the scalp and bones. At night it becomes worse. It is only relieved by powerful anodynes (morphia), and yields to treatment with mercurials and iodides. The insomnia is not necessarily due to headache, as the latter may be absent and insomnia be marked at the same time. Some degree of mental dulness accompanies the headache, and may eventually terminate in stupor or even coma. A condition of cerebral neurasthenia marked by apathy, disinclination for work study or social enjoyment, slowness of thought, irritability of disposition, and impairment of memory, may last for some time. Attacks of vertigo, transitory losses of speech and of muscular power, may also characterise this stage. The general health also begins to fail. The skin and mucous membranes exhibit anæmia and an earthy pallor, appetite fails, strength grows less, and weight is lost. Ptosis and pupillary disturbances are valuable clinical indications which mark this stage. Fever is ordinarily absent. Symptoms of imminent thrombosis or hæmorrhage may appear in middle-aged subjects. If gummata exist local symptoms may preponderate according as the tumour is large or situated in certain areas or tracts of the brain, as e.g. the kinæsthetic and the other sensorial areas. Limited palsies, epileptiform attacks, and apoplectiform seizures mark the presence of gummata or advancing endarteritis. In subjects with a predisposition to insanity, mania, acute mental confusion, acute hallucinatory insanity, stupor, or agitated melancholia may result. Syphilophobia and hypochondriasis arising from the fear of syphilitic infection may have for some time pre-existed in sensitive patients, who as the result of actual infection will then rapidly develop symptoms of mental aberration. The prognosis under such conditions is in most cases grave.

[82] Ibid. (Epitome), 8th July, 1899. [83] Lancet, 9th March, 1901.

